# Citrate versus heparin anticoagulation in continuous renal replacement therapy in small children

**DOI:** 10.1007/s00467-017-3694-4

**Published:** 2017-06-04

**Authors:** Paulien A. M. A. Raymakers-Janssen, Marc Lilien, Ingrid A. van Kessel, Esther S. Veldhoen, Roelie M. Wösten-van Asperen, Josephus P. J. van Gestel

**Affiliations:** 1 Department of Pediatric Intensive Care, Wilhelmina Children’s Hospital, University Medical Center Utrecht, Lundlaan 6, 3584 EA Utrecht, The Netherlands; 2 Department of Pediatric Nephrology, Wilhelmina Children’s Hospital, University Medical Center Utrecht, Utrecht, The Netherlands

**Keywords:** Anticoagulation, Children, Citrate, Heparin, Continuous renal replacement therapy

## Abstract

**Background:**

Citrate is preferred over heparin as an anticoagulant in adult continuous renal replacement therapy (CRRT). However, its potential adverse effects and data on use in CRRT in infants and toddlers is limited. We conducted a prospective study on using citrate in CRRT in critically ill small children.

**Methods:**

Children who underwent CRRT with the smallest filter in our PICU between November 2011 and November 2016 were included. Both heparin and citrate were applied according to a strict protocol. Our primary outcome was circuit survival time. Secondary outcomes were alkalosis, citrate toxicity, and number of red blood cell transfusions.

**Results:**

Heparin was used in six patients (121 circuits, total CRRT time 3723 h). Citrate was used in 14 patients (105 circuits, total CRRT time 4530 h). Median circuit survival time with heparin was 21 h (IQR 14.5–27.5) compared to 45.2 h (IQR 37.5–52.8) with citrate (*p* < 0.001). Actual administered effluent dose compared to prescribed dose was 85% (IQR 69–98%) with heparin compared to 92% (IQR 88–98%) with citrate (*p* = 0.31). No patient treated with citrate developed citrate toxicity. No other differences in electrolytes were found between the two CRRT regimes. In the heparin group, a median of 6.5 units of red blood cells (IQR 1.5–23.8) were given during CRRT, compared to three in the citrate group (IQR 2.0–5.0, *p* = 0.12).

**Conclusions:**

Use of regional citrate significantly prolongs circuit survival time and thereby should increase CRRT efficiency when compared to heparin. In addition, citrate appears safe for CRRT in critically ill small children.

## Introduction

Continuous renal replacement therapy (CRRT) is the most commonly used treatment modality in critically ill patients with severe acute renal injury, diuretic-resistant fluid overload, intoxications, and congenital disorders of metabolism [1, 2]. Efficacy of CRRT is directly related to the running time of the circuit. CRRT interruptions, which are most often caused by extracorporeal circuit clotting, decrease therapy effectiveness, while blood loss, and replacements of the extracorporeal circuit increase costs [2, 3]. Anticoagulation is used to prevent extracorporeal circuit clotting. Heparin is the classic choice for anticoagulation because it is cheap, has an instantaneous antithrombotic effect, and a lot of experience with its use exists. However, since heparin is given systemically in CRRT, it increases the risk of bleeding.

Regional anticoagulation of the CRRT circuit with citrate is an alternative treatment, but it is still not standard of care due to its complexity and concerns regarding complications, such as metabolic alkalosis, accumulation of unmetabolized citrate (citrate toxicity), and, as a consequence, electrolyte disturbances [3]. It has been shown in adults that use of citrate decreases the risk of bleeding when compared to heparin, without a significant increase in incidence of complications [2–5]. Nowadays, citrate is therefore preferred over heparin in critically ill adults. Data from CRRT in adults cannot be automatically applied in children due to the fact that higher circuit extracorporeal volumes relative to patient blood volume and higher relative blood flows in children increase the risks of complications during CRRT. Organ functions like hepatic clearance of citrate are less developed in younger patients, which may increase the risk for alkalosis or citrate toxicity. No CRRT device has been specifically designed and tested for use in children. There are currently no CRRT devices approved by the United States Food and Drug Administration for individuals weighing ≤11 kg, which makes their use off-label in these patients [6].

Only very few studies have described regional citrate anticoagulation in CRRT in critically ill children. We therefore assessed efficacy and complications of CRRT with regional citrate anticoagulation and compared it to heparin anticoagulation in this patient population. Since the main challenges with CRRT are to be found in the smallest patients, we decided to limit our study to CRRT in children weighing up to 15 kg.

## Methods

This study was conducted in a 14-bed mixed medical-surgical pediatric intensive care unit (PICU) of a tertiary university hospital in the Netherlands.

All consecutive patients with a weight below 15 kg, who were admitted to PICU between November 1, 2011, and October 31, 2016, and who received CRRT were included. The responsibility and daily care for acute CRRT was delivered by the intensive care nursing and medical staff in close collaboration with the pediatric nephrologists. Until 2013, heparin was used as anticoagulant in all patients on CRRT. From 2013 onwards, regional citrate anticoagulation is the treatment of choice in all children, unless they already receive heparin for systemic anticoagulation for other reasons.

The following data were collected prospectively from 2011 onwards as part of clinical care for all patients on CRRT in our PICU: demographic data, reason for admission to PICU, Pediatric Index of Mortality (PIM) 2 score [7], reason for CRRT, runtime of the filter, downtime of the circuit, laboratory data (blood gas analyses, serum calcium, potassium, phosphate, sodium, and magnesium levels), number of red blood cells transfused during CRRT, length of PICU stay, duration of mechanical ventilation, and duration of CRRT.

CRRT was performed using the Prismaflex device (Baxter International, Deerfield, IL, USA) including the Prismaflex HF20 circuit (Baxter International, Deerfield, IL, USA). The priming volume of the HF20 circuit is 60 ml. All patients were dialyzed in CVVHDF (continuous veno-venous hemodiafiltration) modality. Venous access was obtained by catheters between 6.5 and 8.0 F depending on age and weight of the child, using the same criteria for patients treated with citrate and heparin anticoagulation. Citrate CRRT was performed with pre-filter anticoagulation and post-filter replacement fluid. Anticoagulation was performed using a commercially available citrate solution (Anticoagulant Citrate Dextrose A solution (ACDA), Baxter International, Deerfield, IL, USA). A calcium-free dialysis solution was used (Prism0cal B22, Baxter International, Deerfield, IL, USA). As post-filter replacement fluid, we used either a phosphate-containing solution (Phoxilium, Baxter International, Deerfield, IL, USA) or a phosphate-free solution with a low or normal potassium content (Prismasol 2 or 4, Baxter International, Deerfield, IL, USA), depending on the potassium level of the patient.

Citrate was started at a dose of 3.0 mmol/l of blood flow. The flow of the citrate solution was coupled to the blood flow and adapted automatically by the software of the Prismaflex device. The citrate dose was modified, if needed, to obtain a circuit ionized calcium level between 0.25 and 0.45 mmol/l (post-filter sample). Post-filter replacement flow and dialysate flow were adjusted to obtain a total effluent dose of 1200 ml/m^2^/h. Before extracorporeal blood was reinfused into the patient, a calcium solution (containing 0.113 mmol Ca/ml <10 kg and 0.225 mmol Ca/ml >10 kg) was continuously infused to neutralize citrate effects. Calcium compensation was defined as the percentage of supplemented calcium compared to calcium loss and citrate dosage. Calcium solution flow was adapted automatically by the software of the Prismaflex device, based on the citrate dose, dialysate flow, replacement fluid calcium content, and type of calcium solution used. Systemic ionized calcium level was maintained in the normal range (between 1.10 and 1.30 mmol/l).

In the heparin anticoagulation protocol, a bolus of 50 UI/kg was administered when connecting the patient to the CRRT circuit, followed by continuous infusion of heparin sodium of 5–15 IU/kg/h to achieve a heparin ratio between 1.7 and 2.2. Replacement fluid was given in pre-dilution mode. Prismasol 2, Prismasol 4, or Phoxilium (Baxter International, Deerfield, IL, USA) were used as dialysate and replacement fluid solutions.

The primary outcome of our study was circuit survival time, defined as time before hemofilter failure (i.e., hemofilter clotting or persistent transmembrane pressures above 200 mmHg, necessitating filter replacement). Secondary outcomes were actual administered effluent dose (prescribed dose minus downtime and technical issues), number of red blood cell transfusions required during CRRT, and the occurrence of acid-base or electrolyte disturbances. Metabolic alkalosis was defined as a pH greater than 7.50, hypokalemia as a serum potassium concentration below 3.5 mmol/l, hypophosphatemia as a serum phosphate concentration below 1.0 mmol/l, hypocalcemia as a serum ionized Ca concentration below 1.10 mmol/l, and citrate toxicity as a total–to-ionized calcium ratio above 2.5.

Children were monitored following a strict protocol. In the heparin group, frequent laboratory tests were done (heparin ratio, serum sodium, potassium, ionized calcium, total calcium, and magnesium levels). During the first 24 h, these tests were done every 3 h. When CRRT and laboratory results were stable, the time interval between tests was increased to 6 h. In the citrate group, the following laboratory tests were done: hematocrit, total platelet count, blood gasses and serum levels of sodium, potassium, ionized calcium, total calcium, phosphate, and magnesium every 3 h during the first 24 h of treatment. When CRRT and laboratory results were stable, the time interval between tests was increased to 6 h.

### Statistical analysis

Statistical analysis was performed using SPSS version 23 (IBM, Armonk, NY, USA). Continuous variables were reported as mean and standard deviation and compared with one-way analysis of variance (ANOVA) followed by Student’s* t* test, or expressed as median and interquartile ranges (IQR) and compared with the Mann–Whitney* U* test, where appropriate. Circuit survival time differences between the two anticoagulation treatments were visualized by Kaplan–Meier plots and tested with a log–rank test. A *p* value <0.05 was considered as statistically significant.

## Results

A total of 4331 children were admitted to PICU between November 1, 2011 and October 31, 2016. Forty of these patients received CRRT, 20 of them had a body weight less than 15 kg (0.46% of all PICU admissions) and were included in the study. Table 1 shows characteristics of the six patients in whom heparin was used (121 circuits, total CRRT time of 3723 h) and of the 14 patients in whom regional citrate anticoagulation was used (105 circuits, total CRRT time of 4530 h). All children were critically ill with no significant differences between PIM2 scores on admission and had acute kidney injury, stage 3 as defined by KDIGO criteria 2012 [8]. Catheter size and location were comparable in both groups (Table 1). All patients were mechanically ventilated during CRRT. Mortality rates were equal in both groups (50%).

**Table 1:** Baseline characteristics of the 20 children <15 kg who received continuous renal replacement therapy (CRRT)

	Heparin (*n* = 6)	Citrate (*n* = 14)	*p* value
Male gender,* n*	4	7	0.64
Age (months), median (IQR)	13 (1–20)	17 (1–33)	0.54
Weight (kg), median (IQR)	9.0 (4.2–11.1)	9.8 (4.4–14.9)	0.51
PIM2 (%, risk of mortality), median (IQR)	17.5 (6.1–40.1)	13.5 (6.7–21.7)	0.29
Reason CRRT (*n*)			
Volume overload	3	9	
oliguria	2	4	
Electrolyte disturbance	1	1	
Admission diagnosis (*n*)			
Toxic shock syndrome	1	0	
SCT oncology	4	7	
Malignant disease (without SCT)	0	4	
nephrotic syndrome	0	1	
post cardiac surgery	1	2	
Dialysis catheter size			0.11
6.5 French	2	8	
8.0 French	4	6	
Dialysis catheter site			0.53
Right jugular vein	4	12	
Femoral vein	1	2	
Right atrium line	1	0	
Length of stay in PICU (days), median (IQR)	26 (23.5–93.5)	15 (9–35)	0.4
Mechanical ventilation (days), median (IQR)	26 (22.5–85.5)	15 (6–24)	0.4
PICU mortality* n* (%)	3 (50%)	7 (50%)	1.0

*PIM* Pediatric Index of Mortality, *IQR* inter-quartile range, *kg* kilogram, *n* number, *SCT* stem cell transplantation, *PICU* pediatric intensive care unit

**Fig. 1:**
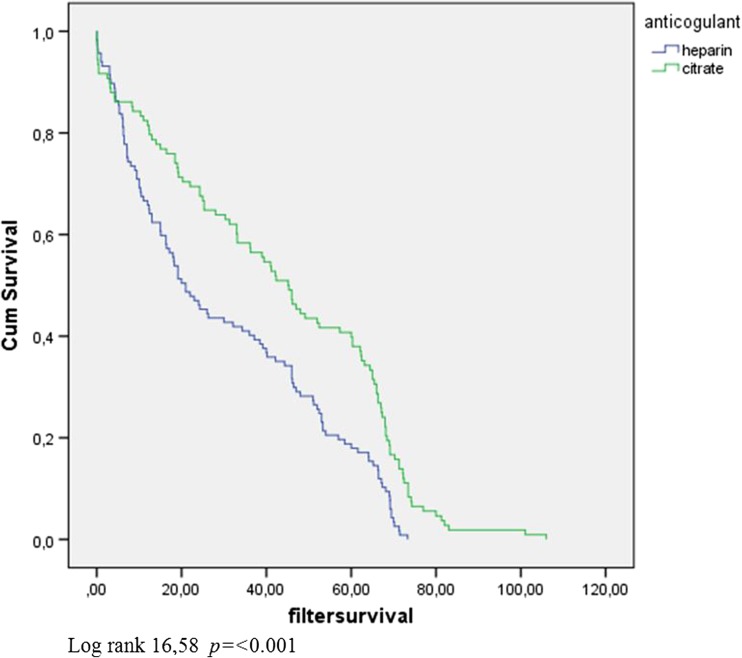
Kaplan–Meier survival curve indicating overall circuit survival time in anticoagulation with heparin or citrate

**Fig. 2:**
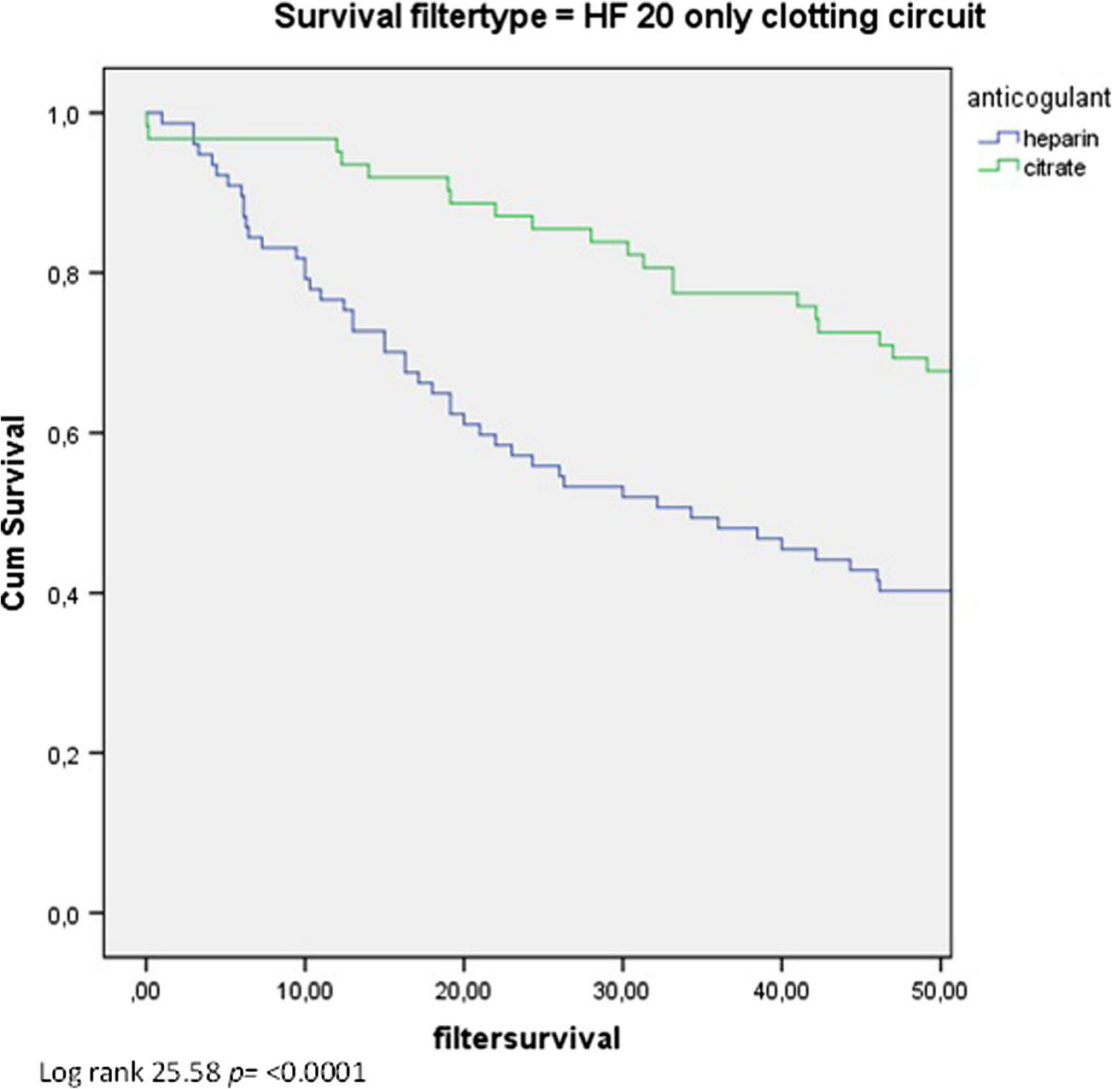
Kaplan–Meier survival curve indicating circuit survival time in the subgroup where only clotted filters were included

Table 2 summarizes CRRT data and laboratory variables in each group. Median circuit survival time with heparin was 21 h (IQR 14.5–27.5) compared to 45.2 h (IQR 37.5–52.8) with citrate (*p* < 0.001) (Fig. 1). In a subgroup analysis, circuit survival times were determined where clotting was the reason for filter failure. In this subgroup, median circuit survival time with heparin was 34 h (IQR 13–60.8) compared to 65.7 h (IQR 42–71.5) with citrate (*p* < 0.001) (Fig. 2).

**Table 2:** Mean continuous renal replacement therapy (CRRT) data and laboratory variables during CRRT with a HF20 filter comparing heparin and citrate in patients <15 kg

	Heparin (*n* = 121)	Citrate (*n* = 105)	*p* value
Time on CRRT, h	3723	4530	
Actual- /prescribed dosage % (IQR)	83 (69–95)	92 (88–98)	0.31
pH (mean, SD)	7.35 (±0.002) *n* = 1387	7.35 (±0.003) *n* = 1505	0.04*
Magnesium (mmol/l), mean (SD)	0.81 (±0.008) *n* = 449	0.80 (±0.005) *n* = 831	0.41
Ionized calcium (mmol/l), mean (SD)	1.24 (±0.003) *n* = 1431	1.21 (±0.003) *n* = 1399	<0.01*
Phosphate (mmol/l), mean (SD)	1.52 (± 0.03), *n* = 209	1.32 (±0.02) *n* = 317	<0.01*
Red blood cells (units) transfused per patient, median (IQR*)*	6.5 (1.5–23.8)	3 (2.0–5.0)	0.12

*IQR* inter quartile range, *SD* standard deviation, *n* numbers

*significant

**Fig. 3:**
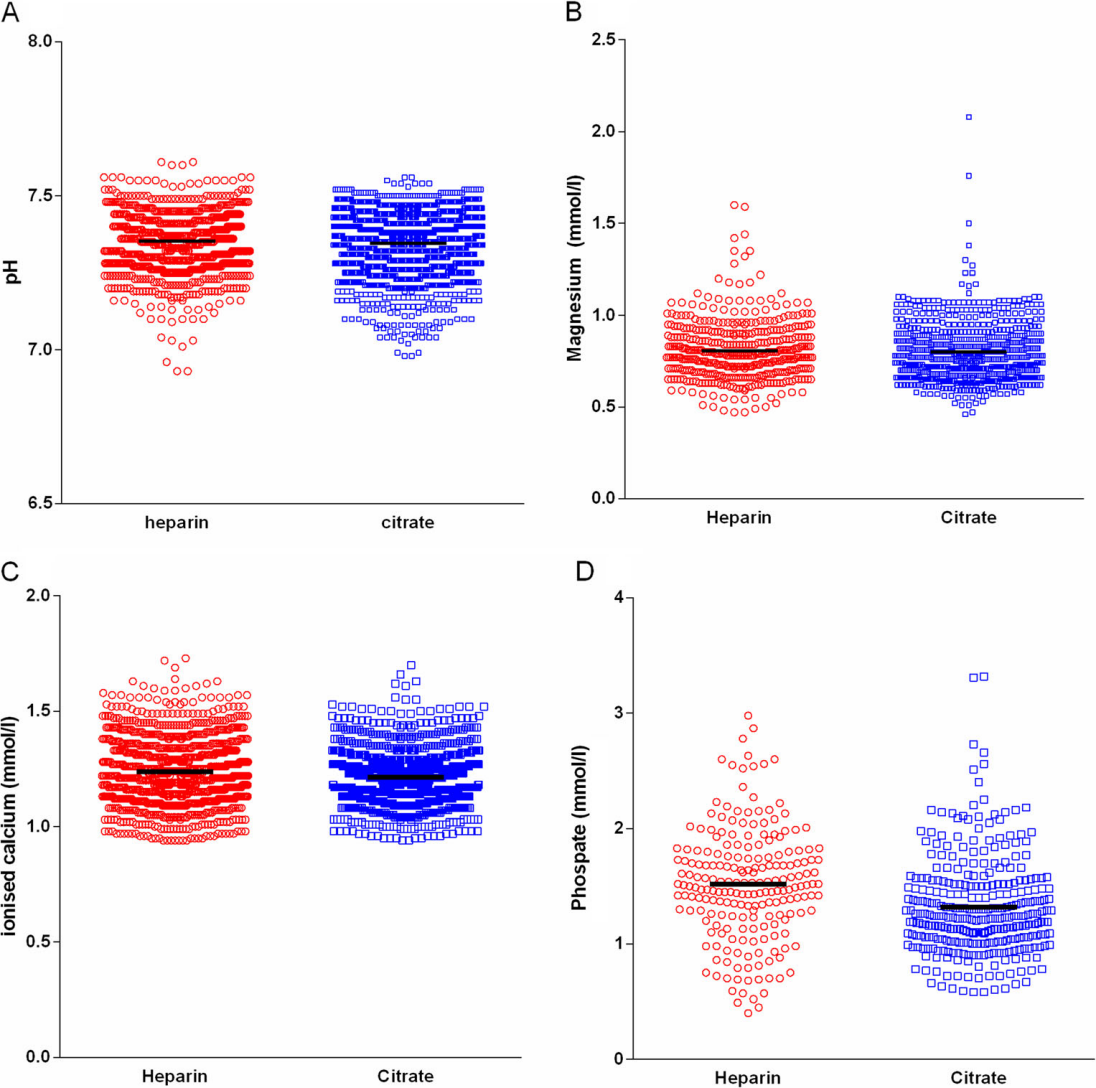
Scatterplot electrolytes during continuous renal replacement therapy (CRRT) treatment

In the citrate group, 3.0% developed a metabolic alkalosis compared with 2.2% in the heparin group (*p =* 0.20). Mean pH in the citrate group was 7.35 ± 0.003 compared with a mean pH of 7.35 ± 0.002 in the heparin group (*p =* 0.04). Serum ionized calcium levels in the citrate group (mean 1.21 ± 0.003) were significantly lower than in the heparin group (mean 1.24 ± 0.003; *p* < 0.001). Nearly all ionized calcium values were in the normal range, and none of the patients suffered from symptomatic hypocalcemia or severe hypocalcemia. Serum phosphate levels were significantly lower in the citrate group (mean 1.32 ± 0.02) than in the heparin group (mean 1.52 ± 0.03; *p* < 0.001). Most values for serum phosphate were in the normal range in both groups, none of the patients developed clinical signs of hypophosphatemia. Serum magnesium levels were equal in both groups. See Table 2 for detailed information and Fig. 3 for graphic plots of electrolytes.

In the heparin group, a median of 6.5 units of red blood cells (IQR 1.5–23.8) were given during CRRT, compared to three in the citrate group (IQR 2.0–5.0; *p* = 0.12).

The actual administered effluent dose compared to the prescribed dose was 85% (IQR 69–98%) in the heparin group compared to 92% (IQR 88–98%) in the citrate group (*p* = 0.31).

Clotting causing hemofilter failure occurred in 42.0% of filters in the heparin group compared to 17.1% of filters in the citrate group (*p <* 0.001; Table 3). In the citrate group, scheduled filter replacement after 72 h was the main reason for hemofilter end-of-survival 40%, compared to 21.4% in the heparin group (*p =* 0.004).

**Table 3:** Reasons for circuit failure in the heparin and citrate group

	Heparin (*n* = 121)	Citrate (*n* = 105)	*p* value
Circuit clotting	51 (42%)	18 (17.1%)	<0.001*
Vascular access malfunction	12 (10%)	9 (8.6%)	0.82
Transport to radiology/operating room	8 (6.6%)	7 (6.7%)	0.98
Switch to other substitution fluid	0	5 (4.8%)	0.02*
Scheduled filter replacement after 72 h**	26 (21.4%)	42 (40%)	0.004*
Technical issues/alarms***	13 (10.8%)	9 (8.6%)	0.66
End of CRRT treatment	6 (5%)	14 (13.3%)	0.03*
Other reasons	5 (4.2%)	1 (0.9%)	0.22

*Significant

**Advised maximum duration of HF20 filter use according to Baxter international, Deerfield, IL, USA

***Incorrect scale balance caused blood pump stop and shutdown of the circuit

## Discussion

Continuous renal replacement therapy requires anticoagulation to prevent filter clotting. The ideal anticoagulant only works in the circuit with minimal effects on patient homeostasis, and should be easy to monitor. Citrate is the preferred anticoagulant in adults, but only very few studies have described its use in children, who may well suffer more from side effects of citrate compared to adults. Our study shows that citrate can be safely applied even in children weighing up to only 15 kg. Using citrate, circuit survival time and CRRT efficiency increased significantly when compared to heparin.

Median circuit survival in our patients was significantly higher for citrate (45.2 h) than for heparin (21 h) (*p <* 0.01), which means that circuit survival time more than doubled with citrate. In a subgroup analysis where we analyzed circuit survival times only in clotted circuits, survival time was nearly twice as long with citrate compared to heparin. Of note, replacement fluid was returned in pre-filter mode in patients on heparin anticoagulation, while it was returned in post-filter mode in regional citrate anticoagulation due to the presence of calcium. In theory, pre-filter replacement (as in use of heparin) may increase the odds for filter survival. Our data support the notion that post-filter replacement does not offset the advantageous effects of regional citrate on filter survival.

Our data on circuit survival times are in line with other studies in pediatric patients (Table 4) [2, 5, 9, 10]. In a prospective crossover study, Zaoral and coworkers showed that citrate provided significantly longer circuit lifetimes compared to heparin [9]. However, patients up to 18 years of age were included in this study. A retrospective study comparing citrate and heparin anticoagulation in critically ill small children found smaller differences in circuit lifetimes between the two groups compared to our findings [5]. In addition, they have analyzed a smaller number of filters. Patients in this study were of about the same age, or even somewhat older than our patients and some of them received CRRT with a larger filter in comparison with the filters used in our study [5].

**Table 4:** Comparable studies

	Age		*N* (total number of inclusions)		*N* (total number of circuits)		Median filter survival time (hours)		Total number of dialysis hours		Filter (m^2^)
	Heparin	Citrate	Heparin	Citrate	Heparin	Citrate	Heparin	Citrate	Heparin	Citrate	
Current study	13 months (1–20)	17 months (1–33)	6	14	121	105	21 (14.5–27.5)	45,2 (37.5–52.8)	3723	4530	HF 20 (0.2 m^2^)
Fernandez et al.^2^	33 months (5.6–84)	36 months (5.6–103)	24	12	71	29	31 (15.5–71)	48 (31–93.7)	NR	NR	HF20 (0.2 m^2^) M60 (0.6 m^2^) M100 (0.9 m^2^)
Soltysiak et al.^5^	15.25 months ± 24	24.15 months ± 28	16	14	43	41	25 (2.25–121)	39.8 (1–171.8)	1398	2379	HF20 (0.2 m^2^) M60 (0.6 m^2^)
Zaoral et al.^9^	84 months (0.5–204)		63 (cross over)		118	118	36 (31–40)	41 (35–52) h*	4219	5162	AV ped (0.2 m^2^) AV400s (0.75 m^2^) AV 600 s (1.4 m^2^)
Brophy et al.^10^	8.6 years ± 7.2		138		442		42.1 ± 27.1	44.7 ± 35.9	18,208		M60 (0.6 m^2^) M100 (0.9 m^2^) HF1000 (1.14 m^2^)

The superscript numbers refer to the literature references

*NR* not reported

The largest study so far from Brophy et al. [10], including 138 patients, found identical filter survival times with heparin and citrate. However, this study included patients up to 25 years old and only a small proportion of their study population consisted of small children. In addition, filter survival times with heparin in this study have not been replicated yet in other studies in children.

Circuit survival time also depends on site and size of vascular catheters used. In both groups, most patients had a catheter in the right jugular vein. More patients in the heparin group had an 8F catheter, which means the observed difference in circuit survival time cannot be explained by a too small catheter size.

The actual delivered effluent dose was higher in the citrate group as compared to the heparin group. The difference did not reach statistical significance, probably because of lack of power in our study. As clearance over the hemofilter is relatively low, dialysis efficacy is mainly determined by treatment time in CRRT. Improving circuit survival time should therefore in the long run improve dialysis dose as well.

The use of citrate anticoagulation is associated with metabolic complications, including possible life-threatening systemic hypocalcemia and metabolic alkalosis [3, 5, 6, 11, 12]. The most important side effect is citrate toxicity when excess citrate binds to free calcium, causing an increase in the ratio of total calcium to ionized calcium. None of our patients developed a symptomatic hypomagnesemia or hypocalcemia, and all pH measurements were in the normal range. In addition, no cases of citrate toxicity were observed. Brophy and coworkers found metabolic alkalosis in 11% of patients and Metha et al. described six of 34 patients (18%) with metabolic alkalosis during citrate anticoagulation [10, 13, 14]. We deliberately used a dialysis solution with a bicarbonate concentration of 22 mmol/l while using regional citrate anticoagulation, to prevent the occurrence of metabolic alkalosis. The data seem to support that this strategy was successful.

Soltysiak et al. found more patients with hyponatremia and hypocalcemia during citrate anticoagulation, but these differences were not statistically significant [5]. Taken together, we are convinced that, when used according to a strict protocol, citrate can be used safely in small patients up to 15 kg.

The use of systemic heparin in critically ill patients may increase the risk of bleeding. Indeed, the need for red blood cell transfusions was higher in the heparin group compared to the citrate group in our study, although not statistically significant. Moreover, these results may have been skewed by one patient in the heparin group, who needed a lot of transfusions because of recurrent blood loss and frequent clotting of the filter. Therefore, we have to interpret our results with caution and cannot conclude from the present findings that citrate limits the need for blood transfusions. Brophy et al. [10] (10% of patients), Fernandez et al. [2] (13% of patients), and the systematic review in adults by Wu et al. [3] have found a significant difference in the proportion of patients experiencing major bleeding with heparin compared to citrate.

Our study has several limitations. Our first patients (2011–2013) received standard anticoagulation with heparin. In April 2013, we started treating patients with regional citrate anticoagulation. Therefore, we cannot rule out an effect of increasing experience with CRRT on improvement of circuit survival time in our study. The number of patients in our study is small. Moreover, the number of patients treated with heparin and citrate are unbalanced, with heparin used in six patients and citrate used in 14 patients. We therefore cannot exclude that some of the patients distorted our findings and have to be careful with drawing firm conclusions. On the other hand, total dialysis time in both groups was long (3723 and 4530 h), and we found results comparable to those in other studies in larger children and in adults. Our findings underscore the advantage of regional citrate anticoagulation in CRRT, even in the most vulnerable population of small children.

## Conclusions

Regional citrate is a safe and effective anticoagulation method for CRRT in very small children, when it is applied following a strict protocol. It significantly prolongs circuit survival time and thereby should increase CRRT efficiency. We did not find any serious adverse effects of regional citrate anticoagulation.
